# A phase I study of the ceramide nanoliposome in patients with advanced solid tumors

**DOI:** 10.1007/s00280-023-04588-7

**Published:** 2023-09-22

**Authors:** Aaron Ciner, Theodore Gourdin, Jeff Davidson, Mylisa Parette, Susan J. Walker, Todd E. Fox, Yixing Jiang

**Affiliations:** 1 Department of Medicine, University of Maryland Greenebaum Comprehensive Cancer Center, Baltimore, MD USA; 2 Department of Medicine, Medical University of South Carolina, Charleston, SC USA; 3 Keystone Nano, Boalsburg, PA USA; 4 Department of Pharmacology, University of Virginia, Charlottesville, VA USA

**Keywords:** Sphingolipids, Ceramides, Cell death, Metabolism, C6-ceramide, Sphingosine-1-phosphate

## Abstract

**Purpose:**

Ceramide is a sphingolipid metabolite that deactivates multiple oncogenic signaling pathways and promotes cell death. In-vivo data demonstrate single-agent anti-cancer activity and enhanced efficacy with combination strategies. This phase I dose-escalation trial evaluated Ceramide nanoLiposomes (CNL) in patients with advanced solid tumors and no standard treatment option.

**Methods:**

The primary objective was to establish the maximum tolerated dose. Secondary objectives included determining the recommended phase II dose, the safety and tolerability, the pharmacokinetic profile and preliminary anti-tumor efficacy.

**Results:**

15 patients with heavily pretreated metastatic disease enrolled. Safety data were analyzed for all patients, while pharmacokinetic data were available for 14 patients. There were no grade 3 or higher treatment-related adverse events. The maximum tolerated dose was not reached and there were no dose-limiting toxicities. The most common grade 1 or 2 treatment-related adverse events included headache, fatigue, constipation, nausea and transaminitis. The maximum concentration and area under the curve increased with dose. Clearance was consistent between doses and was observed mainly through the liver without significant hepatotoxicity. The half-life ranged from 20 to 30 h and the volume of distribution was consistent with a lipophilic drug.

**Conclusions:**

CNL exhibited an encouraging safety profile and pharmacokinetic parameters, with some signals of efficacy including prolonged stable disease in 1 patient with refractory pancreatic cancer. Pre-clinical data indicate potential synergy between CNL and multiple systemic therapies including chemotherapy, targeted therapy, and immunotherapy. Future studies are planned investigating CNL in combination strategies.

**Trial registration:**

This study is registered under ClinicalTrials.gov ID: NCT02834611.

## Introduction

Dysfunctional sphingolipid metabolism is now being recognized as a hallmark of oncogenesis [1–3]. Ceramide, a sphingolipid metabolite is linked to preferential growth arrest and cell death of transformed cells. Mechanistically, ceramide facilitates the dephosphorylation of pro-mitogenic signaling cascades, including AKT, ERK and STAT3, in part through the activation of PKCζ and PP1/PP2A protein phosphatases [4–7]. Chemotherapeutic agents of diverse classes including gemcitabine, irinotecan, and etoposide lead to increased levels of endogenous ceramides and the addition of exogeneous ceramide augments the efficacy of multiple systemic therapy regimens [8, 9]. Relapsed or refractory disease is often associated with upregulation of enzymes that metabolize ceramide to less pro-apoptotic species, including sphingosine-1-phosphate, glycosphingolipids, or sphingomyelin [10]. Thus, restoring elevated endogenous levels of ceramide may be an effective strategy to limit cancer growth.

Multiple pre-clinical studies indicate that targeting sphingolipid metabolism by increasing ceramide levels or inhibiting sphingosine-1-phosphate-mediated cell growth might provide a novel avenue to overcome chemoresistance in advanced malignancies [2, 11]. The administration of exogenous short-chain ceramide (C6-ceramide) is an example of such an approach. Short-chain ceramides are selectively cytotoxic, promoting cell death in several neoplastic cell lines, including breast cancer, melanoma, hepatocellular carcinoma (HCC) and pancreatic cancer [12]. However, its clinical utility is limited by low solubility and poor cell permeability. Polyethylene-glycolated liposomes containing C6-ceramide were developed to overcome these pharmacologic limitations [13]. Ceramide nanoLiposomes (CNL) enhance ceramide’s potency against tumor cells in-vitro and demonstrate anti-cancer activity in animal models of HCC, pancreatic cancer, breast cancer, chronic lymphocytic leukemia, acute myeloid leukemia and natural killer large granulocytic leukemia [5, 10, 14–18]. In-vivo pharmacokinetic and toxicological studies in rats and dogs showed that CNL is well-tolerated [13]. Based on this promising pre-clinical safety and efficacy data, a phase I dose-escalation study was initiated to determine the maximum tolerated dose (MTD) of CNL in patients with advanced solid tumors. Secondary objectives were to determine the recommended phase II dose of CNL, its safety and tolerability, its pharmacokinetic profile and preliminary anti-tumor efficacy.

## Materials and methods

### Patient selection

The trial population included patients with a histologic or cytologic diagnosis of an advanced solid tumor without a curative or standard chemotherapy treatment option. Patients had to be ≥ 18 years of age, have an Eastern Cooperative Oncology Group performance status ≤ 2 and have a life expectancy ≥ 12 weeks. Eligibility required adequate hepatic, renal, and bone marrow function and a washout period of at least 4 weeks (6 weeks for mitoxantrone or mitomycin therapy). Patients were excluded if they had uncontrolled central nervous system (CNS) disease, a primary CNS malignancy, leptomeningeal disease, symptomatic heart failure, myocardial infarction within the prior 6 months, or a positive test for HIV, hepatitis B surface antigen or hepatitis C.

### Treatment plan

This was a multi-center trial performed at the University of Maryland Greenebaum Comprehensive Cancer Center, the University of Virginia Emily Couric Cancer Center, and the Medical University of South Carolina Fred Hollings Cancer Center. This study was approved by the institutional review board at each participating site and conducted in accordance with the ethical standards established in the Declaration of Helsinki.

CNL was administered intravenously in a 250 mL bag of normal saline solution over approximately 2 h. It was given twice weekly on Mondays and Thursdays continuously until disease progression or unacceptable toxicity. One cycle was defined as 4 weeks of therapy. To minimize the risk of an infusion reaction observed with other lipid formulations, premedication with dexamethasone (20 mg), diphenhydramine (25 mg) and famotidine (20 mg) was given intravenously 30 min prior to each CNL infusion.

The study used an accelerated titration design to minimize patient exposure to subtherapeutic dose levels and allow rapid dose escalation. The initial dose level (dose level 1) was 36 mg/m^2^ and was determined from the MTD in canine studies. If there was no grade 2 toxicity suspected to be related to the investigational drug after 1 cycle, the next patient would enter at dose level 2. Upon the first instance of grade 2 toxicity of any type suspected to be related to the investigational drug, the cohort was to be expanded to use a traditional 3 + 3 design for that dose level. The MTD was defined as the dose level at which no more than 1 participant had a dose-limiting toxicity (DLT). This dose would then be the recommended phase II dose. Toxicities were graded according to the National Cancer Institute's Common Terminology Criteria for Adverse Events version 4.0.

### Safety assessments

Clinical history, vital signs, and physical exam were performed weekly for the initial 8 weeks and then monthly. Complete blood counts and serum chemistries were performed weekly, and lipid profile and urinalysis were done monthly. Electrocardiogram was done at baseline, prior to the 1st and 2nd infusion and then every 4 weeks. Any hematologic or non-hematologic toxicity excluding alopecia that was ≥ grade 3 and suspected to be related to the investigational drug was considered a DLT. In addition, grade 3 nausea, vomiting, diarrhea or stomatitis of less than 5 day duration was not considered a DLT. A CNS toxicity ≥ grade 2 was considered a DLT.

### Tumor response assessment

Anti-tumor activity was based on imaging and clinical assessments at baseline, every 2 cycles, and at the end of the study. Radiographic response assessment was based on Response Evaluation Criteria in Solid Tumors (RECIST version 1.1). Only those patients who had measurable disease present at baseline, received at least one cycle of therapy, and had their disease re-evaluated were considered evaluable for response.

### Pharmacokinetics and metabolism

Pharmacokinetic (PK) samples were collected prior to the 1st, 2nd, 4th, and 8th infusions of Cycle 1. In addition, samples were taken at 30 min, 1 h, 1.5 h, 2 h, 3 h, 4 h, 6 h, 24 h, and 48 h after the 1st infusion and at 2 h and 24 h after the 2nd infusion. Maximum concentration (C_max_), half-life (T_1/2_) and clearance of CNL were determined based on PK analysis. AUC was determined using the linear trapezoidal method from 0 to 48 h (AUC_0-48 h_). The PK samples were evaluated by the University of Maryland’s School of Pharmacy for C6-ceramide content in plasma. Plasma was spiked with the internal standard C6-ceramide-13C2,D2 and subjected to base hydrolysis and organic extraction. Extracts were run on a Waters Acquity using a Phenomenex Kinetic HILIC 2.6 μm, 4.6 × 100 cm column with an isocratic elution using acetonitrile:water 50:50% v/v with 0.1% formic acid at 0.4mL/minute and 30 °C. Eluate was analyzed by an inline Water TQ-S mass spectrometer by multiple reaction monitoring. C6-ceramide levels were quantified with an external calibration curve and non-compartmental PK analyses performed using Phoenix WinNonlin software. C6 ceramide metabolism was evaluated by the University of Virginia Cancer Center’s Lipidomic and Metabolomic Shared Resource as described elsewhere [18].

### Statistical analysis

The analytic data set included only patients who received any treatment concordant with the study protocol. The distribution of the DLT was estimated in the search for the MTD. All patients were evaluable for toxicities. Adverse events categorized as possibly, probably, or definitely related to treatment were assessed.

## Results

### Patients

15 eligible patients were enrolled on study. Baseline characteristics of all patients are outlined in Table 1. There were 7 male patients and 8 females. 80% were White and 20% were African-American. 1 patient identified as Hispanic/Latino. The ages of participants ranged from 44 to 83 years with a median age of 58. Patients in this study had metastatic disease and were heavily pre-treated with a median of 4 prior lines of systemic therapy. All patients had received prior surgery and 87% (13/15) had received prior radiation therapy.

**Table 1:** Patients baseline characteristics

	*n*	%
Gender		
Male	7	47
Female	8	53
Race		
White	12	80
Black	3	20
Ethnicity		
Hispanic/Latino	1	7
Non-Hispanic/Latino	14	93
Age		
Range	44–83	
Median	58	
Prior therapy		
Prior surgery	15	100
Prior radiation therapy	13	87
Prior systemic therapy	14	93
Prior lines of therapy		
Range	0–20	
Median	4	
Cancer subtypes		
Colorectal	4	27
Breast	2	13
Lung	2	13
Pancreatic	1	7
Leiomyosarcoma	1	7
Anaplastic thyroid	1	7
Adenoid cystic	1	7
Adrenocortical	1	7
Urothelial	1	7
Leydig cell	1	7

### DLTs, MTD and adverse event profile

Patients received CNL per the dosing schema outlined in Table 2. Dose level 1 started at 36 mg/m^2^ and was increased per protocol to 323 mg/m^2^. There were no DLT nor MTD identified.

**Table 2:** Dosing schema

Dose level	CNL dose	Enrolled	Evaluable DLT	DLT
1	36 mg/m^2^	3	3	0
2	54 mg/m^2^	4	4	0
3	81 mg/m^2^	3	3	0
4	122 mg/m^2^	1	1	0
5	183 mg/m^2^	2	2	0
6	215 mg/m^2^	1	1	0
7	323 mg/m^2^	1	1	0

Adverse events (AE) were determined in all patients who received at least 1 dose of study drug (Table 3). All patients experienced at least 1 treatment-emergent AE (TEAE) during the study, and 47% (7/15) experienced at least 1 grade ≥ 3 TEAE. Any-grade treatment-related AE (TRAE) were experienced by 53% (8/15). *Importantly, there were no grade* ≥ *3 TRAE*. 20% (3/15) experienced a serious adverse event (SAE), but none were considered treatment-related. The most common clinical TRAE were headache in 20% (3/15) and constipation, nausea and fatigue each present in 13% (2/15) of patients. Other clinical TRAE present in 7% (1/15) included chills, diarrhea, facial edema, distal neuropathy, abdominal pain, bone pain and facial flushing. The most common laboratory TRAE were increased aspartate aminotransferase (AST) in 20% (3/15), and increased alanine aminotransferase (ALT) and alkaline phosphatase (AlkPhos) each in 13% (2/15). Other laboratory TRAE present in 7% (1/15) included thrombocytopenia, hypoalbuminemia and hypocalcemia. All TRAE were either grade 1 or 2 in severity and none led to dose reduction or discontinuation of the study drug.

**Table 3:** Adverse events

Any-grade TEAE *n* = *15*	Grade ≥ 3 TEAE	Any-grade TRAE	Grade ≥ 3 TRAE
15/15 (100%)	7/15 (47%)	8/15 (53%)	0/15

Clinical TRAE ≥ 10% patients	Frequency *N*	%	Severity
Headache	3	20	Grade 1
Constipation	2	13	Grade 1–2
Nausea	2	13	Grade 1
Fatigue	2	13	Grade 1–2

Laboratory TRAE ≥ 10% patients	Frequency *N*	%	Severity
↑ AST	3	20%	Grade 1
↑ ALT	2	13%	Grade 1
↑ AlkPhos	2	13%	Grade 1

### Pharmacokinetics

**Fig. 1:**
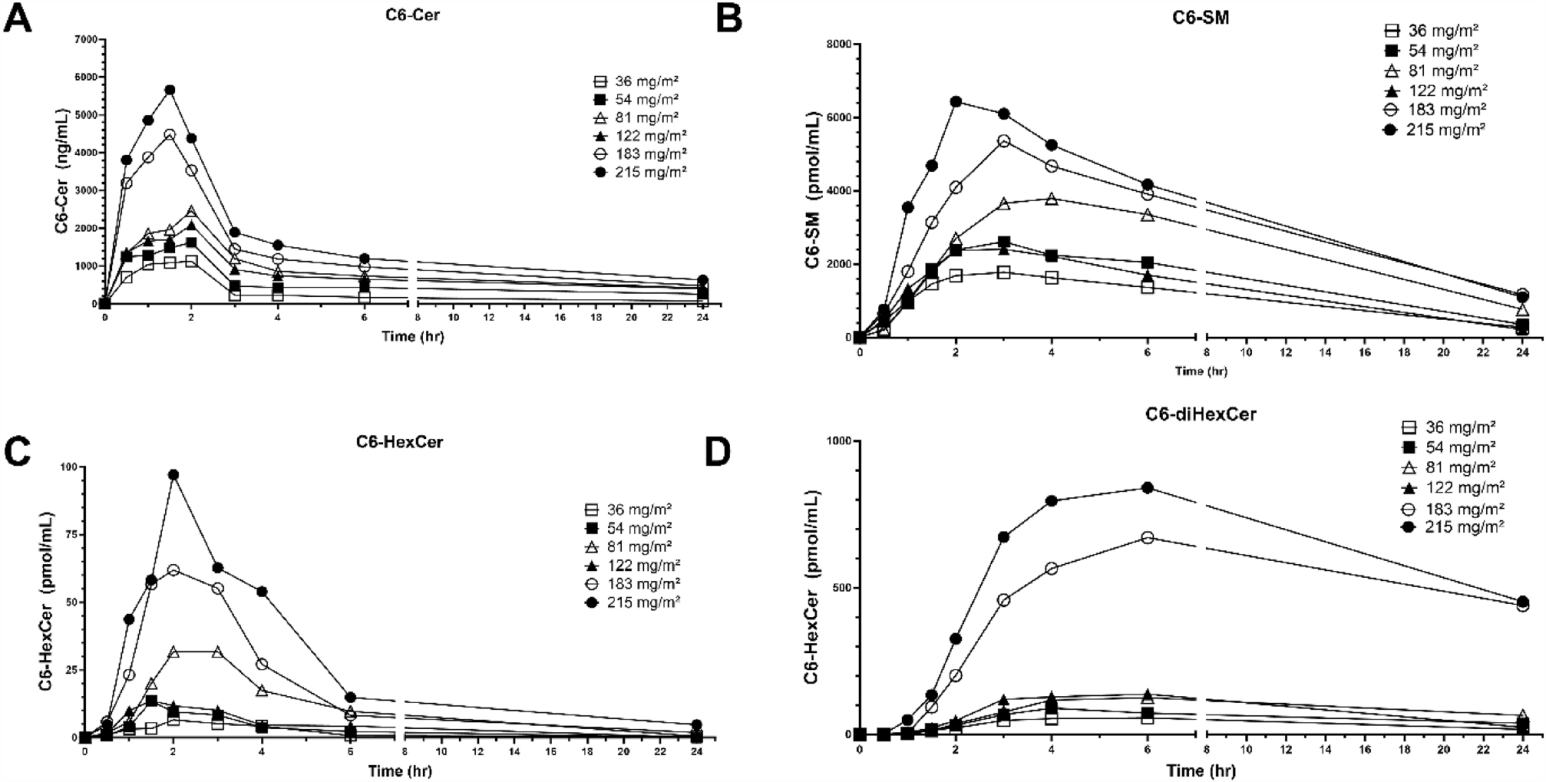
Ceramide and Metabolite Concentrations. This is a graphical representation of the mean doses for C6-ceramide and ceramide metabolites over a 24-h period after administration of CNL. Future studies may shed light on the predictive relevance of ceramide metabolites on clinical outcomes

PK data was available and analyzed for 14 patients and is outlined in Table 4. C_max_ and area under the concentration–time curve (AUC) both increased with dose with *r*^2^ for C_max_ of 0.9126 and for AUC of 0.8393. The small number of patients in each cohort might have contributed to a degree of  variability in the dose linear relationship. Clearance and volume of distribution were consistent between doses. Pre-clinical data indicate that C6-ceramide is preferentially cleared by the liver [13, 19] T_1/2_ ranged from 20 to 30 h and the steady state volume of distribution (Vss) was consistent with a lipophilic drug. The ceramide plasma concentration, which correlates with pre-clinical efficacious dose levels of 36 mg/kg, using allometric scaling, is 108 mg/m^2^ [13, 20]. Importantly, ceramide concentrations above this threshold were achieved without DLT. Targeted LC/MS/MS revealed that a portion of exogenous C6-ceramide was metabolized into C6-sphingomyelin (SM), C6-hexosylceramide (HexCer), and C6-dihexosylceramide (diHexCer) (Fig. 1B–D). C6-ceramide-1-phosphate was not detected. Of note, PK samples for the subject who received 323 mg/m^2^ were lost during shipping.

**Table 4:** PK Parameters

Dose (mg/m^2^)	36	54	81	122	183	215
PK Parameters			Mean Values			
AUC (ng*hr/mL)	6842 ± 676	22,176 ± 14,937	26,018 ± 3080	23,550	34,837 ± 2328	44,657
C_max_ (ng/mL)	1200 ± 285	1759 ± 946	2462 ± 826	2089	4481 ± 573	5663
CL (ml/hr)	4045 ± 992	3666 ± 2491	2341 ± 540	3688	4200 ± 220	3585
V_ss_ (mL)	99,533 ± 52,132	83,716 ± 41,041	74,565 ± 8721	133,714	109,759 ± 13,869	114,396
T_1/2_ (hr)	19.4 ± 8.5	22.7 ± 11.6	25.5 ± 6.5	28.4	21.4 ± 2.7	26.7

### Anti-tumor activity

13 patients were evaluable for response to CNL (Table 5). These patients represented a range of tumor types with refractory metastatic disease. Patients received a median of 15 doses of CNL. No complete or partial responses were observed. 38% (5/13) of participants demonstrated stable disease at 8 weeks and 1 patient with pancreatic cancer showed disease stability for greater than 4 months.

**Table 5:**
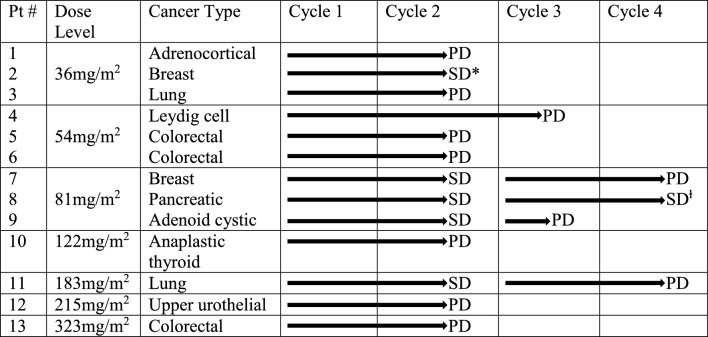
Anti-Tumor Activity

Radiographic response was determined based on RECIST v1.1

*Patient withdrew consent to continue on study

SD continued > 16 weeks until radiographic progression confirmed at 24 weeks

## Discussion

This phase I study of CNL in patients with advanced and refractory solid tumors demonstrated reasonable safety and tolerability with no clinically significant TRAE. No DLT was observed and a MTD was not reached. The human data in this study is consistent with our pre-clinical animal data including a long T_1/2_, large AUC and volume of distribution with minimal toxicity [13]. Of note, the AUC and C_max_ increased with CNL dose without adverse effects. The trial was stopped at a dose of 323 mg/m^2^ due to allowable deliverable volumes. While steady state volume of distribution values were consistent with a lipophilic drug formulation, leaner patients appeared more likely to respond with stable disease compared to those with a higher body mass index. This may reflect distribution and metabolism within adipose tissues and may require further evaluation to determine if dosing regimens should be altered based on body habitus. Pre-clinical data in a rat model using CNL show extensive tissue distribution and indicate that preferential clearance is by the liver [19]. We now also demonstrate that metabolism of C6-ceramide to other C6-metabolites can be detected in the circulation, which may be attributed to metabolizing enzymes in the bloodstream, liver or through interaction with hematopoietic cells. The levels of C6-SM and C6-HexCer appear to peak with C6-ceramide, whereas generation of the higher ordered glycosphingolipid, C6-diHexCer, may be slower to generate (see Fig. 1). Future clinical studies may determine if these profiles reflect changes in efficacy. The twice weekly dosing was determined from pre-clinical experiments in rodents and beagles to achieve steady state levels, where the T_1/2_ was calculated at approximately 20 h. Of note, based upon the lack of significant adverse events, the FDA allowed transition from a 3 + 3 strategy back to 1 patient per dose at 122 mg/m^2^, which reduced the time to study completion, but also limited the total number of patients enrolled.

CNL was developed to synergize with standard-of-care (SOC) approaches based on strong pre-clinical combinatorial activity [10, 21, 22]. CNL can reduce pro-survival gene products such as survivin or MCL-1, induced by standard chemotherapeutic and targeted agents [6, 23]. Moreover, numerous SOC therapeutics can induce enzymatic cascades to increase ceramide formation [8]. Recent in-vivo data also supports an immunomodulatory role for CNL in counteracting the suppressive microenvironment and possible synergy with checkpoint blockade [24]. Thus, it was encouraging to see some degree of efficacy as monotherapy, with 38% of patients demonstrating stable disease in 4 distinct cancer subtypes. The fact that no clinically significant toxicities were noted across 10 different cancers also bodes well for continued evaluation in combination strategies. As a MTD was not reached and efficacy signals were seen beginning at 81 mg/m^2^, we envision a phase II adaptive trial design where CNL is evaluated at 81 mg/m^2^ or 121 mg/m^2^ combined with immunotherapy or cytotoxic chemotherapy. Due to potential synergy, we may be able to use lower doses of standard therapeutics to obtain desired effects or resurrect previous lines of therapy in patients who have become resistant to SOC.

While there are several nanoliposomes or nanoparticles developed as therapeutics, including Doxil (Doxorubicin), DaunoXome (Daunorubicin), Depoct (Cytarabine), Marquibo (Vinblastine), Onivyde (Irinotecan) and Abraxane (Paclitaxel), there are no nanoscale drug delivery systems for bioactive lipids such as ceramides. While the preclinical development of CNL has been described, there are certain distinct engineered parameters that allow for an improved pharmacokinetic and pharmacodynamic profile [13]. For example, CNL is designed to deliver bioactive ceramide via inter-bilayer movement instead of membrane fusion or active receptor targeting, which directs ceramide to the plasmalemmae instead of exosome/lysosome processing [19]. In addition, the dual pegylation strategy used to manufacture CNL assures both long-term shelf-life (2 years) and biological stability [13]. Finally, all patients were pre-treated with anti-histamines and steroids as a precaution, since many nanoliposomes can induce an anaphylactic reaction, and both CNL and the ghost liposomes induced transient local erythema and edema and changes in heart rate and blood pressure readings in beagle dogs [13]. Importantly, with administration of pre-medication, no evidence of hypersensitivity including anaphylaxis was seen for patients on this study.

In sum, CNL exhibited an encouraging safety profile, as well as a long T_1/2_, and large AUC and volume of distribution with some signals of efficacy including prolonged stable disease in 1 patient with pancreatic cancer. These phase I results along with strong pre-clinical data indicating synergy with systemic agents including chemotherapy, targeted therapy and immunotherapy support further investigation of CNL in combination strategies.

## Data Availability

The data sets generated during the current study and subsequently analyzed are available from the corresponding author on reasonable request.
